# Efficacy of cabergoline therapy in patients with non-functioning pituitary adenomas: A single center clinical experience

**DOI:** 10.20945/2359-3997000000495

**Published:** 2022-06-27

**Authors:** Guadalupe Vargas-Ortega, Baldomero González-Virla, Lourdes Balcázar-Hernández, Rocío Arreola-Rosales, Francisco Javier Benitez-Rodríguez, Blas López Félix, Moisés Mercado

**Affiliations:** 1 Unidad de Investigaciones Endocrinas Hospital de Especialidades Centro Médico Nacional Siglo XXI Ciudad de México Mexico Servicio de Endocrinología/Unidad de Investigaciones Endocrinas, Hospital de Especialidades, Centro Médico Nacional Siglo XXI, Instituto Mexicano del Seguro Social (IMSS), Ciudad de México, México; 2 Instituto Mexicano del Seguro Social Centro Médico Nacional Siglo XXI Hospital de Especialidades Ciudad de México Mexico Departamento de Patología, Hospital de Especialidades, Centro Médico Nacional Siglo XXI, Instituto Mexicano del Seguro Social (IMSS), Ciudad de México, México; 3 Instituto Mexicano del Seguro Social Centro Médico Nacional Siglo XXI Hospital de Especialidades Ciudad de México Mexico Servicio de Neurocirugía, Hospital de Especialidades, Centro Médico Nacional Siglo XXI, Instituto Mexicano del Seguro Social (IMSS), Ciudad de México, México

**Keywords:** Nonfunctioning pituitary adenomas, dopamine agonists, cabergoline, dopamine receptors

## Abstract

**Objective:**

To evaluate the response to cabergoline (CBG) treatment in patients with non-functioning pituitary adenomas (NFPA).

**Subjects and methods:**

Retrospective, single tertiary care center study. A total of 44 patients were treated with 3 mg/week of CBG, 32 after surgical treatment (transsphenoidal surgery [TSS] in 27 and TC in 5 patients) and 12 as primary therapy. Mean age was 59.2 ± 12 years and 23 (52.2%) were women. Response to therapy was ascertained by serial magnetic resonance imaging. The median duration of CBG therapy was 30 months (IQR 24-48). Response to CBG therapy was defined as a greater than 20% reduction in tumor size and volume.

**Results:**

A significant reduction in tumor size was documented in 29 patients (66%), whereas in 11 patients (25%) the tumor increased in size and in 4 (9%), it remained stable. Significant tumor shrinkage was documented in 4 (33.3%) of 12 patients treated primarily and in 23 (71.8%) of those treated secondarily. The three-year progression-free survival was 0.61.

**Conclusion:**

Cabergoline therapy is effective in reducing tumor growth in over two thirds of patients with NFPA, however 16% of patients will escape to this beneficial effect and will require alternative forms of treatment to halt tumor progression.

## INTRODUCTION

Nonfunctioning pituitary adenomas (NFPA) represent one of the most common types of pituitary tumor and are not associated with a hormonal hypersecretion syndrome ( 1 ). NFPA comprise a heterogeneous group of tumors that frequently follow an indolent course, however 5%-10% of cases exhibit an aggressive behavior, characterized by rapid growth, invasiveness and early recurrence ( 1 , 2 ). Transsphenoidal surgery (TSS) is the treatment of choice for NFPA however, a non-negligible proportion of patients are left with tumor remnants that require the use of adjuvant therapies ( 3 , 4 ). Adjuvant radiotherapy is usually considered for large tumor remnants or recurrent and/or aggressive adenomas, however, the development of side effect such as hypopituitarism, optic neuropathy and neurocognitive dysfunction, have limit its use ( 3 - 5 ). The majority of NFPA are of gonadotrope differentiation and express somatostatin and dopamine receptors ( 6 ). In contrast to functioning adenomas whereby hormone production by the tumor is used to monitor the response to pharmacological therapy with either somatostatin analogs (SSA) or dopamine agonists (DA), in NFPA the only means to ascertain the response to these medications is the documentation of tumor size reduction by magnetic resonance imaging (MRI) ( 1 , 2 ). This is one of the reasons why there are very few published studies evaluating the response to pharmacological therapy in NFPA. The purpose of this study was to evaluate the effect of cabergoline, a D2R agonist, in a heterogenous group of patients with NFPA diagnosed and followed at a single large tertiary care center.

## SUBJECTS AND METHODS

We retrospectively evaluated all patients diagnosed with a NFPA who were treated primarily or adjunctively with CBG at our center between 2014 and 2020. Patients treated primarily had declined surgery, in the majority of instances out of religious beliefs that made them refuse blood transfusions. Patients treated adjunctively had been subjected to pituitary surgery (transsphenoidal microscopic surgery in 27 patients and transcranial surgery in 5 patients) and were all left with a visible tumor remnant by MRI. Patients who had no visible tumor remnant on postoperative MRI and those who had received radiation therapy were excluded from the analysis, as were those who were treated with SSA. All participating subjects signed the corresponding informed consent, and the protocol was approved by our local Ethics and Scientific Committees.

The diagnosis of NFPA was established on clinical grounds based on the absence of a hormonal hypersecretion syndrome, the variable presence of compressive signs and symptoms such as headache and visual field abnormalities, in the setting of a pituitary adenoma demonstrated by MRI. Patients were started on cabergoline 0.5 mg three times a week (1.5 mg per week) and the dose was increased by 0.5 mg every 3-6 months until achieving a tumor reduction or reaching 3 mg per week. Those patients whose tumors increased in size had their cabergoline discontinued.

MRI was performed after transsphenoidal surgery (TSS), and within three months before CBG treatment by a single, blinded neuroradiologist. Follow up MRI was done every 6-12 months while the patients were being treated with CBG. Changes in remnant size were ascertained by measuring the antero-posterior, transverse and cephalo-caudal diameters and by calculating tumor volume, according to the modified De Chiro-Nelson formula ( 7 ). Patients were thus categorized as having tumor remnant re-growth or reduction, when a greater than 20% increment or reduction in volume were documented on MRI.

Immunohistochemistry was carried out using specific antibodies against anterior pituitary hormones (GH, PRL, TSH, βLH, βFSH and ACTH) and the three main transcription factors (POU1F1, T-Pit and SF-1).

Hormonal measurements were carried out using different commercially available immunoassays. Central hypocortisolism was defined by a 7:00 h cortisol below 5 µg/dL. Central hypothyroidism was diagnosed when the free T4 was below 0.6 ng/dL, along with a low or inappropriately normal TSH. Central hypogonadism was defined by a total testosterone below 250 ng/dL or an estradiol below 20 pg/mL, along with low or inappropriately normal serum LH and FSH. GH deficiency was diagnosed based on a low, age-adjusted IGF-1 measurement.

### Statistical analysis

Quantitative variables are presented either as means with standard deviations (SD) or as medians with interquartile ranges (IQR), according to their distribution. Data distribution was determined by means of the Kolmogorov-Smirnov test. Quantitative variables were analyzed using student T, Mann-Whitney U, or Wilcoxon tests, whereas for qualitative variables we used either X^2^ or exact Fisher tests. Survival curves were plotted using Kaplan-Meier analysis, considering the time elapsed between the date of diagnosis and the date of death or last follow-up. Cox proportional hazard analysis was used for multivariate analysis of competing risks. A p < 0.05 was considered statistically significant. As statistical software, we used SPSS version 17 (SPSS Inc) and STATA version 11.2 (StataCorp).

## RESULTS

A total of 44 patients participated in the study and their basal characteristics are shown in Table 1 . Mean age was 59.2 ± 12 years and 23 (52.2%) were women. The most common complaints were headaches (63.6%) and visual field abnormalities (72.7%). Oculomotor paralysis and intracranial hypertension were each present in one patient. In only one patient was the NFPA found incidentally. Anterior pituitary hormone deficiencies were found as follows: ACTH deficiency in 12 (27.2%), TSH deficiency in 25 (56.8%) LH/FSH deficiency in 14 (31.8%), GH deficiency in 13 (29.5%). Panhypopituitarism, defined by the presence of two or more pituitary hormone deficiencies was found in 9 (20.4%) patients. Based on immunohistochemistry using specific antibodies against anterior pituitary hormones and transcription factors, 62% of the patients harbored gonadotrophinomas and 38% null cell adenomas. Twelve patients received CBG as primary treatment and 32 after TSS (42% one surgery, 33% two surgeries, 3% 3 or more surgeries). The median time between the last surgery and the initiation of CBG was 22 months (IQR 15-52). The median duration of CBG therapy was 30 months (IQR 24-48).

**Table 1:** Patients characteristics at baseline

Age (years)		59.5 ± 12
Gender; n (%)		Female: 23 (52)
		Male: 21 (48)
Headache, n (%)		28 (63.6)
Visual field defect, n (%)		32 (72.7)
Oculomotor palsy, n (%)		1 (2.2)
Intracranial hypertension, n (%)		1 (2.2)
Pituitary hormone deficiencies *		
	Hypothyroidism, n (%)	25 (56.8)
	Hypocortisolism, n (%)	12 (27.2)
	Hypogonadism, n (%)	14 (31.8)
	Hyposomatotropism, n (%)	13 (29.5)
	Panhypopituitarism, n (%)	9 (20.4)
Previous treatment; n (%)		
	Surgery	32 (73%)
	Primary treatment with cabergoline	12 (27%)

* At diagnosis in the 12 patients who were treated primarily and after pituitary surgery in the 22 patients who were treated adjunctively.

Overall, during the follow up period a significant reduction in tumor volume (>20%) was documented in 29 patients (66%), whereas in 11 patients (25%) the tumor increased in size and in 4 (9%), it remained stable. Seven of 44 (16%) patients showed an initial reduction in tumor volume but subsequently the adenoma grew again, therefore they were categorized as non-responders in the final analysis. Among the 12 patients treated primarily, a significant reduction in tumor volume was demonstrated in 4 (33.3%), whereas in 5 (41.6%) the tumor remained stable and in 3 (25%) it increased in size ( Table 2 ). Of the 32 patients treated secondarily after surgery, 23 (71.8%) significantly decreased their tumor volume, in 7 (21.8%) the lesion remained stable and in 2 (6.25%) it increased in size.

**Table 2:** Individual tumor volume outcome of the 12 patients treated primarily with cabergoline (CBG) (*last available measurement while on CBG)

Patient	Age	Sex	Cavernous Sinus invasion	Tumor volume mm^3^		Status
				Pre-CBG	On CBG*	
1	50	M	No	10195	13659	Growth
2	71	M	No	2028	2028	Stable
3	46	F	No	678	778	Stable
4	76	F	No	1949	1714	Stable
5	80	F	Yes	16933	16900	Stable
6	44	F	No	1291	2198	Growth
7	54	M	Yes	9538	523	Reduction
8	64	M	No	653	100	Reduction
9	73	M	Yes	5610	6939	Growth
10	66	F	Yes	2395	2763	Stable
11	41	M	Yes	10257	8164	Reduction
12	75	F	Yes	691	523	Reduction

Mean cephalo-caudal, transverse and anterior-posterior tumor diameters were 24.9 ± 10.7 mm, 26.2 ± 13.3 mm and 23.3 ± 10.1 mm, respectively immediately prior to the initiation of CBG. Cavernous sinus invasion was documented in 27 patients (61.3%). The mean cephalo-caudal and transverse tumor diameters, but not the anterior-posterior diameter decreased significantly after CBG treatment ( Table 3 ). Median tumor volume decreased from a baseline of 5,179 mm^3^ (IQR 2074-14383) to 4,983 mm^3^ (IQR 1649-10707) (p = 0.01) after CBG treatment ( Table 3 ). Median percent reduction in tumor size was 36% (IQR 20%-65%) ( Table 3 ). Imaging evidence of pituitary hemorrhagic necrosis, not present in the pre-CBG MRI, was documented in 3 patients, but none of these patients presented with clinically apparent pituitary apoplexy (i.e, loss of consciousness, hemodynamic collapse, cranial nerve abnormalities). CBG was generally well-tolerated, with only occasional reports of nausea and mild gastrointestinal discomfort.

**Table 3:** Tumor size and volume before and after cabergoline treatment (*last available measurement while on CBG)

	At the beginning of cabergoline treatment	On cabergoline treatment*	p
Mean cephalocaudal diameter (mm)	24.9 ± 10.7	20.3 ± 8.9	0.001
Mean transverse diameter (mm)	26.2 ± 13.3	22.8 ± 11.3	0.001
Mean anterior-posterior diameter (mm)	23.3 ± 10.1	23.1 ± 11.1	0.93
Median tumor volume (mm^3^)	5179 (2074-14383)	4983 (1649-10707)	0.01

Tumor progression-free median survival was 48 months (95% CI 24-48) for the primary treatment group, and 30 months (95% CI 24-36) for the secondary treatment group (p = 0.02). Actuarial tumor progression-free survival of the general group at 36 months follow up was 0.5 (95% CI 0.26-0.69, p = 0.01). Tumor progression-free survival was 0.51 (95% CI, 0.16-0.78, p = 0.01) for patients treated primarily and 0.67 (95% CI 0.39-0.84, p = 0.01) for those treated after surgery. Upon multivariate analysis age, gender, cavernous sinus invasion, tumor size, tumor volume, among others, could not confidently predict tumor progression.

## DISCUSSION

In this single center study, we have shown that cabergoline treatment results in tumor size reduction or stabilization in over two thirds of our patients with NFPA, followed for more than two years. Although they do not produce a hormonal hypersecretion syndrome, NFPA frequently invade neighboring structures that are not surgically accessible and therefore their complete resection is not feasible and frequently tumor remnants are left after TSS ( 1 , 2 ). Adenoma recurrence rate can be as high as 30% in some series, and although these recurrent or persistent tumors are usually slowly growing, most of these patients will eventually need several pituitary surgeries and/or an adjunctive form of therapy ( 3 , 4 ). Radiation therapy has extensively been used in these cases and is reasonably successful although it has several side effects which limit its use, such as the development of pituitary hormone deficiencies ( 5 ).

Unlike hormone-producing pituitary adenomas like somatotropinomas, corticotropinomas and prolactinomas, whereby treatment with DA and SSA have proven effective ( 8 - 10 ), the pharmacological treatment of NFPA is not well established. Yet, NFPA are known to express both, dopamine as well as somatostatin receptors and thus, at least in theory, should be biologically capable of responding to SSA and DA ( 6 , 11 ). Pivonello and cols. evaluated 9 NFPA patients with significant remnants after pituitary surgery who were treated with cabergoline for one year ( 11 ). In 5 of these patients, whose tumors all expressed D2R mRNA, a significant tumor shrinkage was documented ( 11 ). Of the remaining 4 patients whose tumors did not shrink with cabergoline treatment in only one was the expression of D2R mRNA demonstrated ( 11 ). Subsequent studies have looked at the relationship between the expression of D2R by immunohistochemistry and the response to cabergoline treatment ( 12 ). Over 90% of patients who respond to this dopamine agonist have evidence of D2R protein expression in their adenomas however, the majority of non-responders tumors also immunostain for this receptor ( 12 ). Thus, using D2R immunohistochemistry to predict a favorable response to cabergoline treatment is currently not useful.

The fact that no major controlled studies have been undertaken to explore the efficacy of DA in these patients is largely due to the lack of a measurable biomarker that could be used to ascertain the response to therapy. Thus, the evaluation of the response to pharmacological therapy in NFPA relies on imaging methods to monitor tumor size. Table 4 summarizes the few studies that have looked into the efficacy of cabergoline treatment in patients with NFPA. Lohman and cols. retrospectively evaluated 13 patients who were treated with cabergoline after pituitary surgery and found that in more than half of them the tumor remnant decreased in size by 10% or more ( 13 ). Greenman and cols., retrospectively evaluated 55 NFPA patients who were treated with cabergoline preventively after TSS and 24 patients who received the dopamine agonist after their adenoma had recurred ( 14 ). Using a 2-mm change in adenoma size as the response criterion, 38% of the adenomas were considered to have decreased in size ( 14 ). More recently, Batista and cols., published the only prospective study that had evaluated the efficacy of cabergoline in this setting ( 12 ). These authors randomized over 100 patients to either cabergoline treatment or conservative wait-and-see non-intervention ( 12 ). After two years of follow up, 28.8% of patients receiving cabergoline and 10.5% of non-treated controls had decreased their adenoma size by more than 25% ( 12 ). Interestingly, in this study in 74% of the patients who did not receive cabergoline their tumors did not grow and remained stable, which to some extent overshadows the efficacy of the dopamine agonist ( 12 ). In our study, we found a higher response rate than in the aforementioned studies, yet our 3-year tumor progression free survival was lower, indicating an escape from the beneficial effects of cabergoline in 16% of patients. Ours is the only study that includes patients who had not been subjected to pituitary surgery and were primarily treated with cabergoline. Interestingly, such primarily treated patients and those treated after TSS appeared to respond equally well to cabergoline.

**Table 4:** Summary of the main studies evaluating the response to cabergoline therapy in patients with NFPA

Series	N/Design	Criteria	Tumor size status			Progression-Free Survival
			Reduction	Growth	Stable	
Lohman, 2001	13/Retrosp	10% change	53.8%	7.7%	38.4%	--
Greenman, 2016	55/Retrosp	2 mm change	38%	13%	49%	0.88 (5 yrs)
Batista, 2019	59/Prosp	25% change	28.8%	5%	66%	0.94 (1 yr)
Current study	44/Retrosp	20% change	66%	25%	9%	0.67 (3 yrs)
	12 primary					
	32 secondary					

The main limitation of our study is its retrospective nature and perhaps the lack of D2R immunohistochemistry. Still, our study reports real-life data, obtained out of the context of a control study. We conclude that over two-thirds of patients with NFPA respond to CBG treatment by reducing tumor remnant size, however, a substantial proportion escapes to this beneficial effect and will require an alternative form of therapy.

## References

[1] Molitch ME (2017). Diagnosis and treatment of pituitary adenomas: a review. JAMA.

[2] Mercado M, Melgar V, Salame L, Cuenca D (2017). Clinically non-functioning pituitary adenomas: Pathogenic, diagnostic and therapeutic aspects. Endocrinol Diabet Nutr.

[3] Yavropoulou MP, Tsoli M, Barkas K, Kaltsas G, Grossman A (2020). The natural history and treatment of non-functioning pituitary adenomas (non-functioning PitNETs). Endocr Relat Cancer.

[4] Vargas G, Gonzalez B, Ramirez C, Ferreira A, Espinosa E, Mendoza V, Guinto G, Zepeda E, Mercado M (2015). Clinical characteristics and treatment outcome of 485 patients with non-functioning pituitary macroadenomas. Int J Endocrinol.

[5] Ntali G, Capatina C, Fazal-Sanderson V, Byrne JV, Cudlip S, Grossman AB, Wass JA, Karavitaki N (2016). Mortality in patients with non-functioning pituitary adenoma is increased: systematic analysis of 546 cases with long follow-up. Eur J Endocrinol.

[6] Ramírez C, Cheng S, Vargas G, Asa SL, Ezzat S, González B (2012). Expression of Ki-67, PTTG1, FGFR4, and SSTR 2, 3, and 5 in nonfunctioning pituitary adenomas: a high throughput TMA, immunohistochemical study. J Clin Endocrinol Metab.

[7] Ertekin T, Acer N, Turgut AT, Aycan K, Ozçelik O, Turgut M (2011). Comparison of three methods for the estimation of the pituitary gland volume using magnetic resonance imaging: a stereological study. Pituitary.

[8] Mercado M, Espinosa E, Ramírez C (2016). Current status and future directions of pharmacological therapy for acromegaly. Minerva Endocrinol.

[9] Gadelha MR, Vieira L (2014). Efficacy of medical treatment in Cushing’s disease: a systematic review. Clin Endocrinol (Oxf).

[10] Wang AT, Mullan RJ, Lane MA, Hazem A, Prasad C, Gathaiya NW (2012). Treatment of hyperprolactinemia: a systematic review and meta-analysis. Syst Rev.

[11] Pivonello R, Matrone C, Filippella M, Cavallo LM, Di Somma C, Cappabianca P (2004). Dopamine receptor expression and function in clinically nonfunctioning pituitary tumors: comparison with the effectiveness of cabergoline treatment. J Clin Endocrinol Metab.

[12] Batista RL, Musolino NRC, Cescato VAS, da Silva GO, Medeiros RSS, Herkenhoff CGB (2019). Cabergoline in the Management of Residual Nonfunctioning Pituitary Adenoma: A Single-Center, Open-Label, 2-Year Randomized Clinical Trial. Am J Clin Oncol.

[13] Lohmann T, Trantakis C, Biesold M, Prothmann S, Guenzel S, Schober R (2001). Minor tumor shrinkage in non functioning pituitary adenomas by long-term treatment with the dopamine agonist cabergoline. Pituitary.

[14] Greenman Y, Cooper O, Yaish I, Robenshtok E, Sagiv N, Jonas-Kimchi T (2016). Treatment of clinically nonfunctioning pituitary adenomas with dopamine agonists. Eur J Endocrinol.

